# CD40 ligation-induced ERK activation leads to enhanced radiosensitivity in cervical carcinoma cells via promoting autophagy: CD40-ERK enhances cervical cancer cell radiosensitivity via autophagy

**DOI:** 10.3724/abbs.2024229

**Published:** 2024-12-17

**Authors:** Baocai Liu, Yadong Zhang, Quan Wang, Qian Wang, Zhixin Wang, Li Feng

**Affiliations:** 1 Department of Radiation Oncology China-Japan Union Hospital of Jilin University Changchun 130033 China; 2 Department of Urology China-Japan Union Hospital of Jilin University Changchun 130033 China

**Keywords:** CD40, autophagy, radiosensitivity, cervical cancer

## Abstract

CD40, a member of the tumor necrosis factor (TNF) receptor superfamily, plays an important role not only in the immune system but also in tumor progression. CD40 ligation reportedly promotes autophagy in immune cells. However, the effects of CD40 ligation on autophagy and its mechanism in solid tumor cells are still unclear. In this study, we find that CD40 ligation promotes autophagosome formation and consequently promotes autophagic flux in cervical cancer cells. Mechanistically, this effect relies on ERK contributing to CD40 ligation-induced ATG13 upregulation by p53. Furthermore, we demonstrate that CD40 ligation-induced autophagy increases the radiosensitivity of cervical cancer cells. Taken together, our results provide new evidence for the involvement of the CD40 pathway in autophagy and radiotherapy in cervical cancer cells.

## Introduction

CD40 is expressed not only on normal B lymphocytes and antigen-presenting cells but also on the surface of epithelioid tumors (cervical cancer, ovarian cancer, lung cancer, bladder cancer, and liver cancer) and hematological tumors [ 1, 2]. CD40L (CD154), a type II membrane protein, is a ligand of CD40 that can be divided into two types, soluble and membrane-bound, both of which can exert their biological effects after binding to CD40 [3]. CD40 can form homotrimers under the stimulation of CD40L or combine with other members of the TNFR family to form heterotrimers. Polymerized CD40 can be autophosphorylated to recruit downstream signal molecules, which activate the PI3K-AKT, Ras-Raf-MEK-ERK and STAT3 signaling pathways to regulate gene expression, thus playing important roles in humoral and cell-mediated immune responses and in the development of tumors. Studies have shown that CD40 activation can inhibit the survival of many kinds of tumor cells, such as B-cell lymphoma, multiple myeloma, bladder cancer, ovarian cancer, breast cancer, skin cancer and cervical cancer cells, and improve the sensitivity of tumor cells to drugs [ 4, 5], but its effect on cancer radiosensitivity is still unclear.

Cervical cancer is the fourth most common cancer among women worldwide, with approximately 604,000 new cases and 342,000 deaths in 2020, 90% of which are in developing countries [6]. At present, approximately 80% of invasive cervical cancer cases require radiotherapy, among which radiotherapy is the main treatment method for IB2-IIA non-surgical patients and IIB-IV patients [7]. Radiotherapy can induce autophagy, which can regulate radiosensitivity. However, the role of autophagy in radiotherapy for cervical cancer is controversial [ 8, 9]. Some reports indicated that increased autophagy has a protective effect on cervical cancer cells. In contrast, other studies showed that the induction of autophagy can increase the radiosensitivity of cervical cancer cells, but the reason for this contradiction is not clear and may be related to the level of autophagy.

Autophagy flux determines the level of autophagy, which consists of the following successive steps: formation of the omegasome from the endoplasmic reticulum, expansion and nucleation of the phagophore into an autophagosome, maturation into amphisomes by fusing autophagosomes with endosomal compartments, and fusion with lysosomes to form autolysosomes [ 10– 13]. In mammals, the initiation of autophagy is controlled by the autophagy-related protein (ATG)1/ULK1 kinase complex, which contains the Ser/Thr kinase ULK1 and the accessory proteins ATG13, FIP200, and ATG101 [ 14, 15]. ATG13 is critical for correctly localizing ULK1 to the pre-autophagosome and for maintaining the stability of the ULK1 protein [16]. ULK1 kinase activity is essential for the subsequent recruitment of the ATG14-containing class III phosphatidylinositol 3-kinase complex, which leads to the enrichment of phosphatidylinositol 3-phosphate (PtdIns3P) in the endoplasmic reticulum and eventually to the recruitment of PtdIns3P-binding proteins, such as the zinc finger, FYVE domain containing 1 (ZFYVE1), to the site of autophagosome generation [ 17– 19]. Two ubiquitin-like conjugation systems, ATG12 and ATG8/LC3B, are required for the expansion and completion of autophagosomes [ 20, 21].

CD40 ligation reportedly promotes autophagy to kill parasites in immune cells [ 22, 23]. However, the role and mechanism of CD40 in the autophagy and radiosensitivity of cervical cancer cells are still unclear.

In this study, we demonstrated that CD40 activation enhances the formation of autophagosomes and autophagic flux by increasing extracellular signal-regulated kinase (ERK)-p53 signaling-mediated upregulation of ATG13 in cervical carcinoma cells. Moreover, the CD40 signaling pathway enhances the radiosensitivity of cervical cancer cells by increasing autophagy level. These findings provide new insight into the involvement of the CD40 pathway in autophagy and radiotherapy in solid cancer cells.

## Materials and Methods

### Cell culture and transfection

Human HeLa and SiHa cells were purchased from the American Type Culture Collection (Manassas, USA) and were grown in RPMI-1640 medium (Invitrogen, Grand Island, USA) and Dulbecco’s modified Eagle’s medium (Invitrogen) supplemented with 10% fetal bovine serum (HyClone, Logan, USA), respectively. The pcDNA3/CD40 plasmid was transfected into HeLa cells using Lipofectamine 2000 (Thermo Fisher Scientific, Waltham, USA). The cells were cultured in medium containing 500 μg/mL G418 (Shenggong Biotechnology, Shanghai, China) for two weeks at 37°C for selection. HeLa cells stably overexpressing CD40 (HeLa/CD40 cells) were treated with 500 ng/mL CD40 ligand (CD40L/CD154) (Cell Signaling Technology, Beverly, USA) for 24 h or pre-treated for 30 min with 10 μM U0126 (Cell Signaling Technology) before being stimulated with CD40L. The stably transfected GFP-LC3B HeLa cell line (HeLa/GFP-LC3B cells) was a kind gift from Yingyu Chen (Peking University, Beijing, China). Autophagy was inhibited by treating cells with 25 μM chloroquine (Sigma-Aldrich, St Louis, USA) for 4 h. Cells were transfected with plasmids using MegaTran 1.0 Transfection Reagent (OriGene, Rockville, USA) and with small interfering RNAs (siRNAs) using Lipofectamine 2000.

### Plasmid construction and siRNA

The GFP-ZFYVE1 and mTagRFP-mWasabi-LC3B plasmids were kindly provided by Yingyu Chen (Peking University). The mCherry-ATG5 and CD40 plasmids were constructed by our laboratory. All plasmids were confirmed by DNA sequencing. siRNAs targeting CD40 were designed and synthesized by Qiagen (Germantown, USA), and siRNAs targeting ATG13 were obtained from GenePharma (Suzhou, China). The siRNA sequences for CD40 and ATG13 are listed in Supplementary Table S1.

### Semiquantitative real-time polymerase chain reaction (RT-PCR)

Total RNA samples from control and CD154-stimulated cells were extracted with TRIzol reagent (Invitrogen, Grand Island, USA). RT-PCR was performed using the ThermoScript RT-PCR System (Invitrogen). In brief, first-strand cDNA was generated using the total RNA in a standard reverse transcriptase reaction using a poly(dT) oligonucleotide as a primer and SuperScript II reverse transcriptase (Invitrogen). The cDNA were then subjected to semiquantitative real-time polymerase chain reaction (RT-PCR) analysis. The primers for key autophagy genes are listed in Supplementary Table S2.

### Western blot analysis

Co-immunoprecipitation assays were performed with control and CD40L-stimulated HeLa/CD40 cells. After 24 h of CD40L stimulation, the cells were harvested in buffer containing 20 mM Tris-HCl (pH 8.0), 150 mM NaCl, 2 mM EDTA, 10% glycerol, 0.5% Nonidet P-40, 1 mM dithiothreitol, 1 mM phenylmethylsulfonyl fluoride, 5 μg/mL leupeptin, 5 μg/mL aprotinin, 5 μg/mL pepstatin, and 1% protease inhibitor cocktail (Roche, Basel, Switzerland). Protein concentrations were determined using a bicinchoninic acid assay kit (Pierce, Rockford, USA). Whole-cell lysates were separated by 10% sodium dodecyl sulfate‒polyacrylamide gel electrophoresis and electrotransferred onto polyvinylidene difluoride membranes (GE Healthcare, Wisconsin, USA). Western blot analysis was performed according to standard protocols [24].

The following antibodies were used: anti-GFP, anti-GAPDH, anti-ATG13, anti-ULK1, anti-p62, anti-ERK, anti-phospho-ERK ^Thr202/Tyr204^ (all from Cell Signaling Technology), anti-LC3B (Sigma-Aldrich), and anti-CD40 (Abcam, Cambridge, USA). The secondary antibodies used included anti-mouse and anti-rabbit DyLight 800- and DyLight 680-conjugated IgGs (Rockland Antibodies & Assays, Limerick, USA). The signals were detected via an Odyssey Infrared Imager (LI-COR Bioscience, Lincoln, USA) or viewed via ECL Plus (GE Healthcare).

### Confocal microscopy

The cells were washed with phosphate-buffered saline, fixed, and permeabilized with 4% paraformaldehyde for 30 min at 4°C. They were then blocked in a solution containing 0.1% Triton X-100 and incubated with the indicated antibodies for 60 min at 37°C. The cells were then washed twice with phosphate-buffered saline and stained with Hoechst 33342 (Cell Signaling Technology) for 10 min before being imaged with an Ultra View VOX confocal laser scanning microscope (Perkin Elmer, Waltham, USA).

### Colony formation assay

The cells at the logarithmic growth phase were seeded into a 6-well plate with a certain number of cells (300 cells/well in the 0 Gy group, 1500 cells/well in the 2 Gy group, 3000 cells/well in the 4 Gy group, 6000 cells/well in the 6 Gy group, and 8000 cells/well in the 8 Gy group), with three replicates in each group. In accordance with the experimental requirements, the cells were irradiated with different doses and cultured for 2 weeks. The conditioned medium (containing 500 ng/mL CD40L) was changed every 3 days to observe the formation of clones. The cells were fixed and stained with 0.5% crystal violet (Beyotime Institute of Biotechnology), and colonies of at least 50 cells were counted by GelCount (Oxford Optronix, Oxfordshire, UK). The adhesion rate was calculated as the number of clones per well in the control group/number of cells inoculated per well, and the survival fraction (SF) was calculated as the number of clones per well in the experimental group/(number of cells implanted per well×adhesion rate)×100%. Survival curves were fitted with a multitarget single-hit model [S = 1 − (1 − e ^−D/D0^) ^N^], where D is the radiation dose and D0 is the average lethal dosage of cells and N is the extrapolation number, via GraphPad Prism 5.0 (GraphPad Software, San Diego, USA).

### Statistical analyses

Data are expressed as the mean ± SEM. Statistical analyses were performed using two-tailed Student’s *t* tests in Prism 5.0 (GraphPad Software). Differences were considered significant if *P* is less than 0.05.

## Results

### CD40 ligation promotes autophagic flux

**Figure 1:**
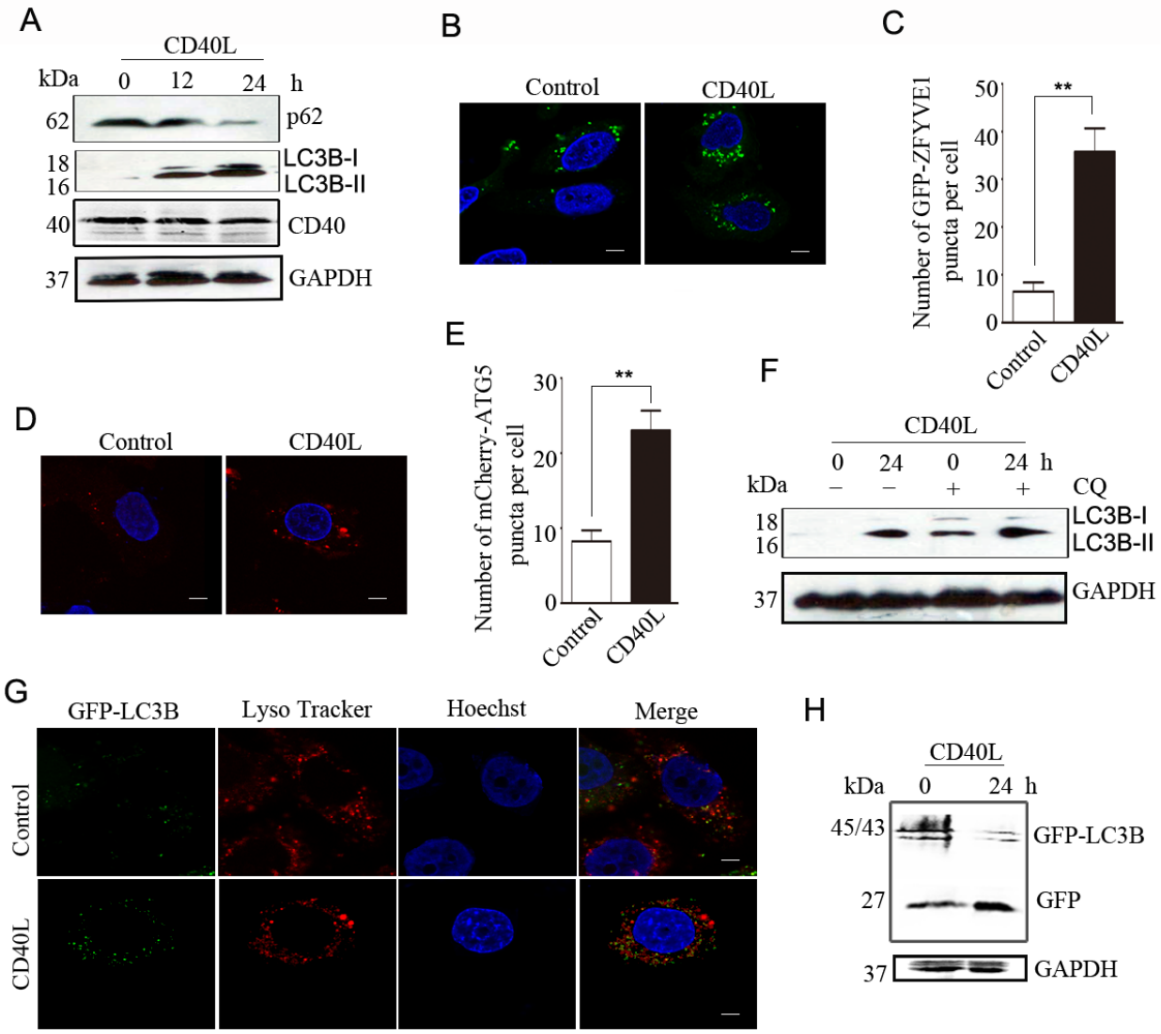
Figure 1 CD40 ligation promotes autophagic flux (A) HeLa/CD40 cells were treated with CD40L for the indicated time. The protein levels of LC3B, p62, and CD40 were detected by western blot analysis. GAPDH was used as the internal standard. (B) HeLa/CD40 cells were transfected with GFP-ZFYVE1 for 12 h and then stimulated with CD40L for 24 h. After fixation, cell images were captured using a confocal microscope. Scale bars: 5 μm. (C) GFP-ZFYVE1 puncta per cell were quantified. The results are expressed as the mean ± SEM of at least 20 cells (**P < 0.01). (D) HeLa/CD40 cells were transfected with mCherry-ATG5 and then treated as described in (A). Images of the cells were captured via a confocal microscope. Scale bars: 5 μm. (E) mCherry-ATG5 puncta per cell were quantified. The results are expressed as the mean ± SEM of at least 20 cells (**P < 0.01). (F) HeLa/CD40 cells were stimulated with CD40L for 24 h and then treated with 25 μM chloroquine (CQ) for 4 h. The level of the LC3B protein was detected by western blot analysis. GAPDH was used as the internal standard. (G) HeLa/GFP-LC3B cells were transfected with a CD40 plasmid. Representative fluorescence microscopy images of the co-localization of GFP-LC3B with LysoTracker Red 24 h after transfection are shown. Scale bar: 5 μm. (H) HeLa/GFP-LC3B cells were transfected with a CD40 plasmid. The level of free GFP was analyzed by western blot analysis. The data are representative of three independent experiments.

To evaluate whether CD40 ligation induces autophagy in cervical cancer cells, we first analyzed the expression of endogenous LC3B-II, a marker of autophagosomes, and the level of p62, an autophagy cargo receptor, in HeLa/CD40 cells by western blot analysis. CD40 ligation increased the conversion of LC3B-I to LC3B-II and decreased p62 expression ( Figure 1A).

To determine the step at which the autophagy process is affected by CD40 ligation in HeLa/CD40 cells, we examined the formation of omegasomes, which can be marked specifically by the endoplasmic reticulum-associated PtdIns3P-binding protein ZFYVE1. HeLa/CD40 cells that were transfected to overexpress GFP-ZFYVE1 were observed under a confocal microscope. CD40 ligation significantly increased the number of GFP-ZFYVE1-labelled vesicles ( Figure 1B,C), suggesting that CD40 ligation enhances the formation of omegasomes.

We subsequently detected the formation of ATG5-labelled membrane structures, which are autophagosome precursors. As shown in Figure 1D,E, CD40 ligation increased the number of membrane-bound mCherry-ATG5 structures in HeLa/CD40 cells. These results indicate that CD40 ligation may enhance autophagosome formation.

To confirm these results, the lysosome inhibitor chloroquine was used to prevent fusion between autophagosomes and lysosomes. In the presence of chloroquine, more LC3B-II accumulated in CD40L-stimulated cells than in control cells ( Figure 1F). These findings indicated that the elevated LC3B-II level driven by CD40 ligation is resulted from increased autophagosome formation.

We then overexpressed CD40 in HeLa/GFP-LC3B cells and used LysoTracker Red to label lysosomes. More GFP-LC3B puncta were observed to co-localize with lysosomes in CD40L-stimulated cells than in control HeLa/GFP-LC3B cells ( Figure 1G), suggesting that CD40 activation may facilitate autophagic flux. After delivery to lysosomes, the GFP-LC3B protein is cleaved, and LC3B is rapidly degraded, while the GFP moiety remains stable [25]. Compared with that in control cells, the free GFP level in HeLa/GFP-LC3B cells was increased by CD40L stimulation, as illustrated by western blot analysis ( Figure 1H). Taken together, these data support the hypothesis that CD40 activation promotes autophagic flux by increasing autophagosome formation.

## Knockdown of *CD40* impairs CD40L-mediated autophagy

**Figure 2:**
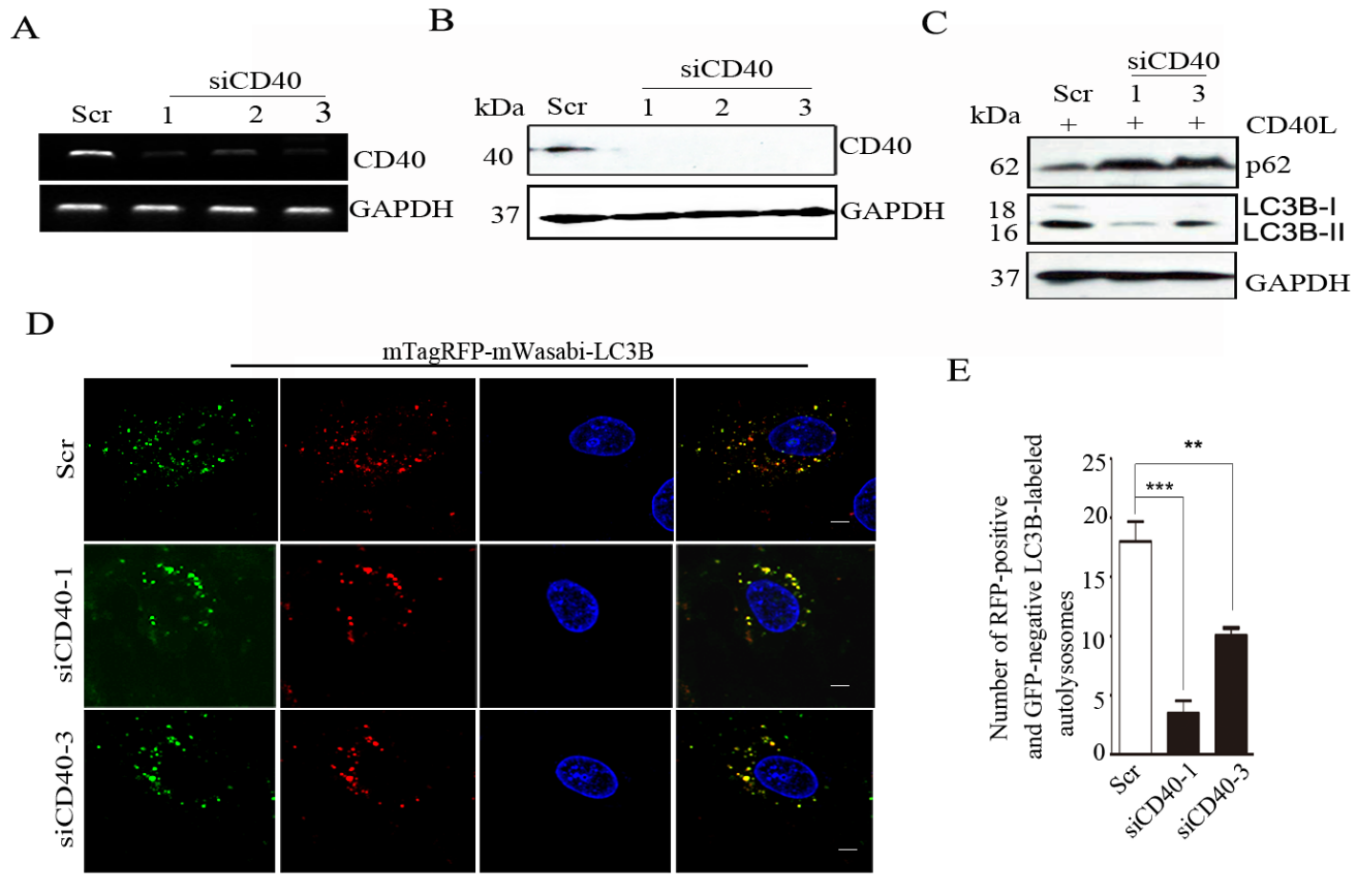
Figure 2 Knockdown of *CD40* impairs CD40L-mediated autophagy (A,B) SiHa cells were transfected with control siRNA (Scr) or siCD40-1, -2, or -3. CD40 knockdown was detected by semi-quantitative polymerase chain reaction (A) and western blot analysis (B). (C) SiHa cells were transfected with Scr or siCD40-1 or -3 and stimulated with CD40L for 24 h. The protein expressions of LC3B and p62 were analyzed by western blot analysis. GAPDH was used as the internal standard. (D) Representative images of mTagRFP-mWasabi-LC3B distribution in CD40L-stimulated SiHa cells co-transfected with mTagRFP-mWasabi-LC3B and Scr or siCD40-1. Scale bar: 5 μm. (E) The number of RFP-positive and GFP-negative LC3B-labelled autolysosomes per cell was quantified. The results are expressed as the mean ± SEM of at least 20 cells (**P < 0.01, ***P < 0.001).

To confirm the effects of CD40 ligation on autophagy regulation, we used *CD40* siRNAs to knock down endogenous *CD40* in SiHa cells. CD40 level was significantly decreased by siCD40-1, -2, and -3 ( Figure 2A,B). CD40 ligation-stimulated LC3B-II expression was determined by western blot analysis and was decreased in *CD40*-knockdown cells compared with that in control cells ( Figure 2C). Consistent with these findings, the p62 level was increased in CD40L-treated *CD40*-knockdown cells ( Figure 2C).

The mTagRFP-mWasabi-LC3B reporter was used to evaluate autophagic flux in *CD40*-knockdown SiHa cells. As shown in Figure 2D,E, *CD40* knockdown decreased the CD40L-induced transition of mTagRFP-mWasabi-LC3B-positive autophagosomes to mTagRFP-positive, mWasabi-negative autolysosomes. Taken together, these results suggest that CD40 activation facilitates autophagy in cervical carcinoma cells.

### CD40 ligation promotes autophagy by upregulating ATG13 expression

**Figure 3:**
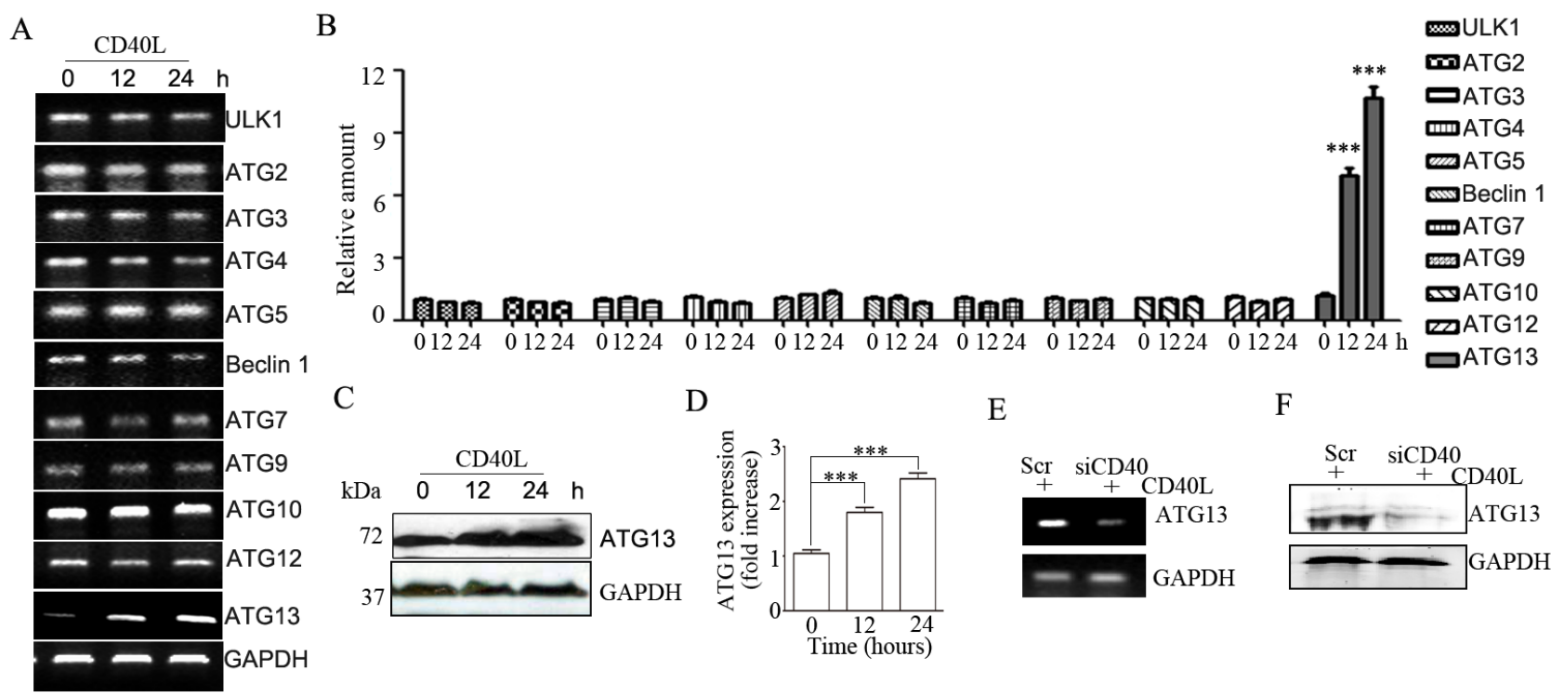
Figure 3 CD40 ligation increases ATG13 expression (A) HeLa/CD40 cells were treated with CD40L for the indicated time. The mRNA expressions of core ATG genes were analyzed using the semi-quantitative polymerase chain reaction. (B) The gray densities of the target bands were analyzed by ImageJ software and normalized to the gray density of GAPDH. The average relative gray density with SEM from three independent experiments is shown (***P < 0.001). (C) The protein expression of ATG13 was analyzed by western blot analysis. (D) The gray densities of the target bands were analyzed by ImageJ software and normalized to the gray density of GAPDH. The average relative gray density with SEM from three independent experiments is shown (***P < 0.001). SiHa cells were transfected with control siRNA (Scr) or siCD40-1 and stimulated with CD40L for 12 h. (E,F) The expression of ATG13 was analyzed by semi-quantitative polymerase chain reaction (E) and western blot analysis (F). GAPDH was used as the internal standard.

The core ATG proteins involved in autophagosome formation are divided into five subgroups: the ATG1/ULK1 protein-kinase complex, the ATG9-ATG2-ATG18 complex, the Vps34-ATG6/Beclin 1 class III phosphatidylinositol 3-kinase complex, and the ATG12 and ATG8/LC3 conjugation systems. Because CD40 activation can affect gene transcription through downstream signaling pathways, we first detected the mRNA expressions of core ATG genes in CD40L-stimulated HeLa/CD40 cells. Among the *ATG* molecules examined, only the transcription of *ATG13* was increased by CD40 ligation ( Figure 3A,B). Western blot analysis demonstrated that CD40 ligation also increased the protein expression of ATG13 ( Figure 3C,D). The effect of CD40 ligation on ATG13 expression was confirmed in *CD40*-knockdown SiHa cells ( Figure 3E,F). These results indicate that CD40 ligation increases ATG13 expression.

**Figure 4:**
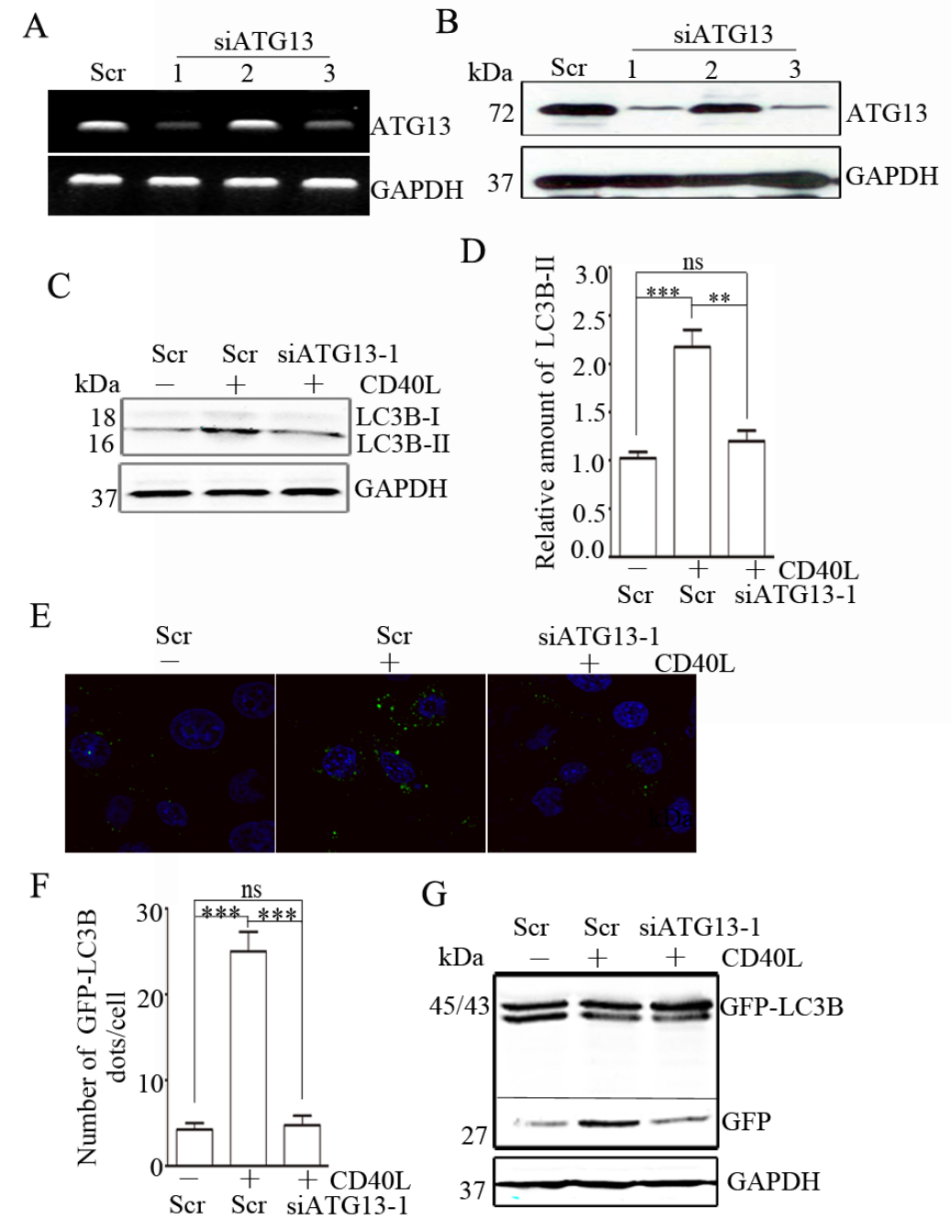
Figure 4 ATG13 mediates CD40 ligation-induced autophagy (A,B) HeLa/CD40 cells were transfected with three distinct ATG13 shRNAs or the matching control non-targeting siRNA (Scr). ATG13 levels were detected by the semi-quantitative polymerase chain reaction (A) and western blot analysis (B). (C) HeLa/CD40 cells were transfected with Scr or siATG13-1. The protein expression of LC3B was analyzed by western blot analysis. GAPDH was used as the internal standard. (D) The gray densities of the target bands were analyzed by ImageJ software and normalized to the gray density of GAPDH. The average relative gray density with SEM from three independent experiments is shown (***P < 0.001). (E) Representative confocal microscopy images of GFP-LC3B distribution are shown. (F) GFP-LC3B puncta per cell were quantified and are expressed as the mean ± SEM of at least 20 cells (***P < 0.001, ns, not significant). (G) The level of free GFP was analyzed by western blot analysis.

To test whether CD40 ligation-stimulated autophagy is dependent on ATG13 expression, we used several *ATG13* siRNAs to knock down endogenous *ATG13* in HeLa/CD40 cells and assessed LC3B-II expression, which revealed that ATG13 level was significantly silenced by siATG13-1 and -3 ( Figure 4A,B). Compared with control siRNA, *ATG13* knockdown decreased CD40 ligation-stimulated LC3B-II expression ( Figure 4C,D). In CD40-overexpressing HeLa/GFP-LC3B cells, knockdown of *ATG13* reversed the CD40 ligation-stimulated formation of GFP-LC3B puncta ( Figure 4E,F) and the increase in free GFP level caused by CD40L stimulation ( Figure 4G). These results suggest that CD40 ligation promotes autophagy by increasing ATG13 expression.

### CD40 ligation increases ERK-p53 signal transduction

**Figure 5:**
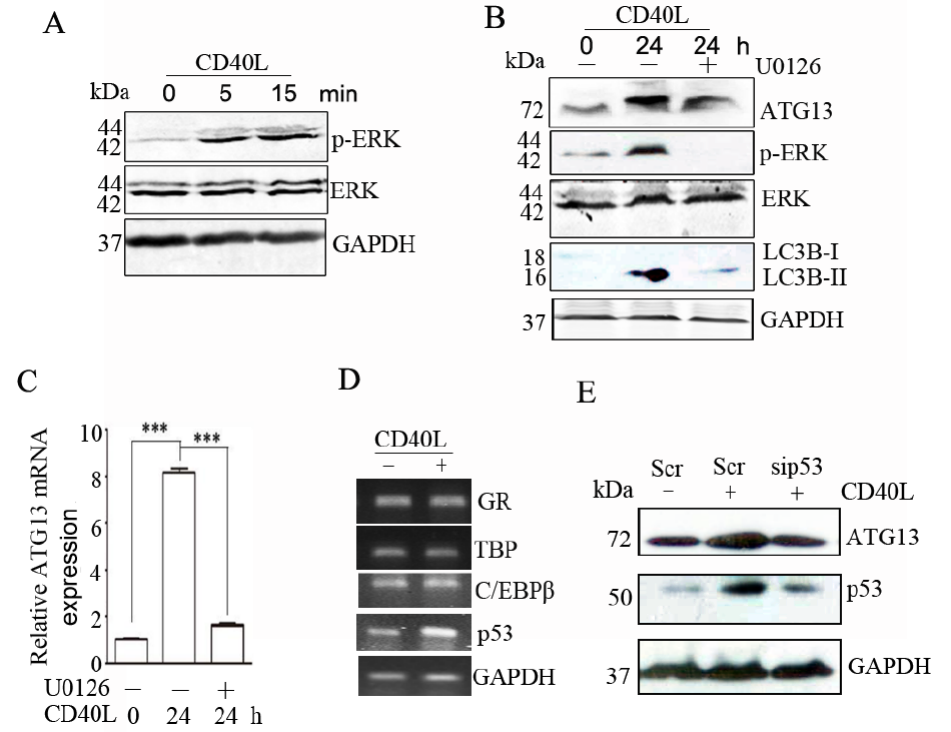
Figure 5 CD40 ligation increases ERK-p53 signal transduction (A) HeLa/CD40 cells were stimulated with CD40L for the indicated time. The levels of phosphorylated and total ERK were detected by western blot analysis. HeLa/CD40 cells were treated with CD40L for 24 h or pre-treated with U0126 for 30 min before being stimulated with CD40L. (B,C) The levels of ATG13, LC3B, phosphorylated ERK, and total ERK were detected by western blot analysis (B), and ATG13 mRNA expression was analyzed using real-time RT-PCR (C). (D) HeLa/CD40 cells were stimulated with CD40L for 12 h, and C/EBPβ, p53, TBP and GR mRNA levels were detected by the semi-quantitative polymerase chain reaction. (E) HeLa/CD40 cells were transfected with Scr or sip53. After 24 h, the cells were treated with CD40L for 12 h, and the protein levels of ATG13 and p53 were detected by western blot analysis. The data are representative of three independent experiments.

CD40 ligation induces mitogen-activated protein kinase signaling pathways to affect the expression levels of many genes [26]. We found that CD40 ligation increased the level of phosphorylated ERK but not p38 in HeLa/CD40 cells ( Figure 5A and Supplementary Figure S1). Importantly, the ERK inhibitor U0126 abrogated the upregulated expressions of ATG13 and LC3B-II induced by CD40 ligation ( Figure 5B,C). These findings suggest that ERK signaling contributes to CD40 ligation-induced autophagy and ATG13 upregulation.

To determine the transcription factor by which CD40-ERK regulates the expression of ATG13, we first predicted the transcription factors in the functional region of the ATG13 promoter by using the UCSC database ( http://genome.ucsc.edu/) and the PROMO database ( http://alggen.lsi.upc.es/). A total of 24 transcription factors were found (data not shown). By reviewing the literature, we further selected the transcription factors regulated by the ERK/MAPK signaling pathway, including C/EBPβ, p53, TBP and GR [ 27- 31], for further study. We found that CD40 ligation increased the transcription level of p53 but not C/EBPβ, TBP or GR in HeLa/CD40 cells ( Figure 5D), which was confirmed in *CD40*-knockdown SiHa cells ( Supplementary Figure S2). Furthermore, consistent with the transcription level, CD40 ligation also increased p53 expression at the protein level ( Figure 5E). Importantly, *p53* knockdown reversed the enhancing effect of CD40 ligation on ATG13 expression ( Figure 5E). These results indicate that p53 increases ATG13 level during CD40-ERK signal transduction.

### CD40 ligation-induced autophagy increases the radiosensitivity of cervical cancer cells

**Figure 6:**
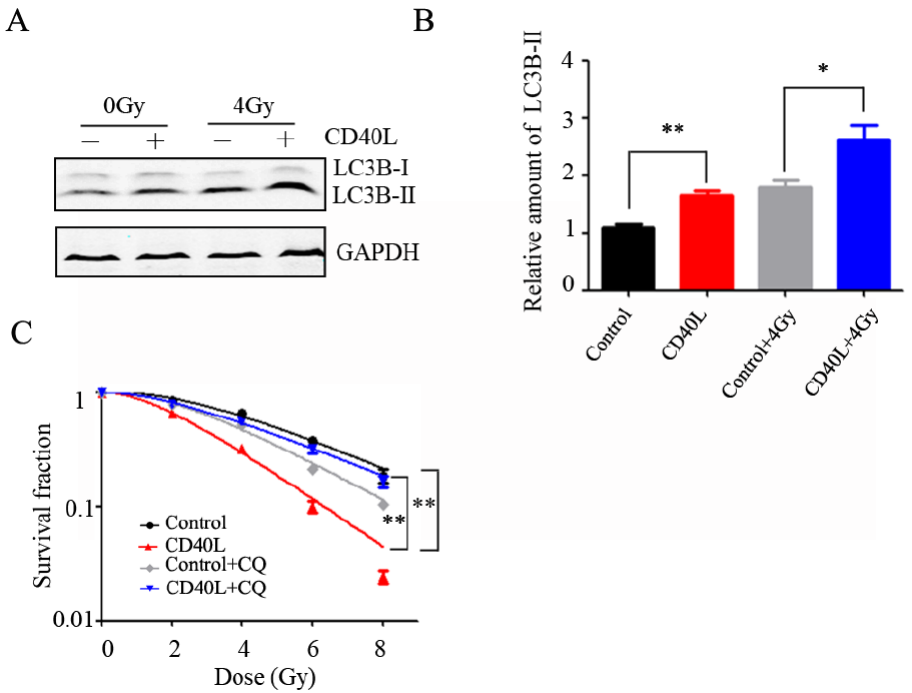
Figure 6 CD40 ligation-induced autophagy increases the radiosensitivity of cervical cancer cells HeLa/CD40 cells were treated with CD40L or 4Gy X-ray alone or in combination for 24 h. (A) The protein levels of LC3B and GAPDH were detected by western blot analysis. (B) The densitometry data normalized to the mean values of three independent experiments via ImageJ software are presented as the mean ± SEM (*P < 0.05, **P < 0.01). (C) Clonogenic survival fraction curves of the control, CD40L, control + CQ and CD40L + CQ groups following exposure to 0, 2, 4, 6, or 8 Gy X-rays (**P < 0.01).

We demonstrated that CD40 activation can increase the level of autophagy in cervical cancer cells. We further detected the effect of CD40 ligation on radiation-induced autophagy by western blot analysis. As shown in Figure 6A,B, CD40 ligation further increased the level of radiation-induced LC3B-II, indicating that CD40 activation can promote radiation-induced autophagy. A recent study showed that high expression of CD40/CD40L is associated with a better prognosis in patients with cervical cancer [32], but whether CD40 activation affects the radiosensitivity of cervical cancer cells is unclear. The survival curve of a multi-target single-hit model simulated from colony formation experiments revealed that CD40 activation increased the radiosensitivity of HeLa/CD40 cervical cancer cells, which was reversed by the autophagy inhibitor CQ ( Figure 6C), and the same effect was observed in SiHa cells ( Supplementary Figure S3), suggesting that CD40/CD40L-induced autophagy enhances the radiosensitivity of cervical cancer cells.

## Discussion

Accumulating evidence suggests that CD40 activation can increase autophagy through different molecular mechanisms to kill *Toxoplasma gondii*. For example, in macrophages, CD40 activation enhances autophagic flux to kill *T*. *gondii* through increasing ULK1 phosphorylation and dissociating Bcl-2 and Beclin 1 [ 22, 23]. However, the functional effect of CD40 activation on autophagy in solid tumor cells is still unclear.

In this study, we used cervical carcinoma cells and demonstrated that CD40 ligation enhanced the formation of autophagosomes, consequently promoting autophagic flux by increasing ATG13 expression. In our experimental system, CD40 ligation increased only the mRNA level of ATG13 among the eleven examined ATG molecules. ATG13 can function as an adaptor through recruiting ULK1, FIP200, and ATG101 to form the ULK1 protein kinase complex. Furthermore, ATG13 is critical for stabilizing the ULK1 protein in 293T, HeLa and mouse embryonic fibroblasts [ 33– 35]. In the present study, we found that CD40 ligation had no effect on the mRNA level of ULK1 in HeLa cells but increased its protein level ( Supplementary Figure S4). These results suggest that CD40 activation may increase the stability of ULK1 by regulating ATG13 expression.

As a tumor suppressor, p53 coordinates a variety of responses, including cell cycle arrest, DNA repair, aging and apoptosis, mainly by regulating the transcription of its target genes [ 36, 37]. Studies have also shown that p53 can induce autophagy directly as a transcription factor of autophagy-related genes. For example, p53 upregulates the transcription of *Cathepsin D*, *TGM2*, *Sestrin1*, *Sestrin2*, and *BNIP3* and further upregulates autophagy [36]. Combined ChIP sequencing and RNA sequencing analysis revealed that p53 binds to many autophagy genes, including *ATG2*, *ATG4*, *ATG7* and *ATG10* [38], but whether p53 affects the autophagy gene *ATG13* has not been reported. In this study, we found that p53, which is downstream of the CD40-ERK signaling pathway, directly upregulated the transcription of *ATG13* to increase autophagy, suggesting that p53 is a transcription factor of *ATG13*, which needs further experimental verification.

The effect of autophagy on the radiosensitivity of cervical cancer has been reported, but the results are inconsistent. For example, radiotherapy can increase the level of autophagy in HTB35 cervical cancer cells, and inhibiting autophagy by knocking down the autophagy-related genes ( *ATG3* and *ATG12*) can increase the sensitivity of these cells to radiotherapy, indicating that radiotherapy-induced autophagy can reduce radiosensitivity [8]. However, recent research has shown that the radiosensitivity of cervical cancer can be increased by photomodulation (PBM) through autophagy pathways, which leads to the induction of apoptosis, increased ROS and damaged DNA [9]. In the present study, activating CD40 signaling enhanced autophagic flux and promoted the radiosensitivity of cervical cancer cells, which was reversed by an autophagy inhibitor. Notably, in our experimental system, autophagy induced solely by radiotherapy played a protective role in cervical cancer cells. In recent years, Dr. Gerwitz and colleagues [39] reported dual functions of autophagy in the response of breast tumor cells to radiation: cytoprotective autophagy with radiation alone and cytotoxic autophagy in radiosensitization by vitamin D3. On this basis, they proposed an attractive theory about the “autophagy switch”, which describes autophagy as a cytoprotective process in irradiated breast tumor cells. However, after increasing autophagy to a certain level, protective autophagy can transform into cytotoxic autophagy, thereby increasing radiosensitivity [40]. As in our experimental system, both cytoprotective and cytotoxic autophagy can occur simultaneously in cervical cancer cells, and the underlying mechanisms deserve further study.

In summary, our study demonstrated that CD40 activation is associated with ATG13 expression and autophagy by increasing the phosphorylation level of ERK in cervical carcinoma cells. In addition, the promotion of ATG13 expression is induced by p53. Importantly, we demonstrated that CD40 ligation-induced autophagy increases the radiosensitivity of cervical cancer cells. These results revealed a novel mechanism and function of CD40 ligation as a positive regulator of autophagy in cervical carcinoma cells, providing new directions for examining the effects of CD40 ligation on autophagy and function in other solid cancer cells.

## Supplementary Data

Supplementary data is available at *Acta Biochimica et Biophysica Sinica* online.

## References

[1] Yan C, Saleh N, Yang J, Nebhan CA, Vilgelm AE, Reddy EP, Roland JT (2021). Novel induction of CD40 expression by tumor cells with RAS/RAF/PI3K pathway inhibition augments response to checkpoint blockade. Mol Cancer.

[2] Yan C, Richmond A (2021). Hiding in the dark: Pan-cancer characterization of expression and clinical relevance of CD40 to immune checkpoint blockade therapy. Mol Cancer.

[3] Ma DY, Clark EA (2009). The role of CD40 and CD154/CD40L in dendritic cells. Semin Immunol.

[4] Li DK, Wang W (2020). Characteristics and clinical trial results of agonistic anti‑CD40 antibodies in the treatment of malignancies (Review). Oncol Lett.

[5] Bereznaya NM, Chekhun VF, Expression of CD40 and CD40L on tumor cells: the role of their interaction and new approach to immunotherapy,
Exp Oncol. 2007, 29: 2–12. https://pubmed.ncbi.nlm.nih.gov/17431381/.

[6] Arbyn M, Weiderpass E, Bruni L, de Sanjosé S, Saraiya M, Ferlay J, Bray F (2020). Estimates of incidence and mortality of cervical cancer in 2018: a worldwide analysis. Lancet Glob Health.

[7] Faye MD, Alfieri J (2022). Advances in radiation oncology for the treatment of cervical cancer. Curr Oncol.

[8] Apel A, Herr I, Schwarz H, Rodemann HP, Mayer A (2008). Blocked autophagy sensitizes resistant carcinoma cells to radiation therapy. Cancer Res.

[9] Djavid GE, Bigdeli B, Goliaei B, Nikoofar A, Hamblin MR (2017). Photobiomodulation leads to enhanced radiosensitivity through induction of apoptosis and autophagy in human cervical cancer cells. J Biophotonics.

[10] Yang Z, Klionsky DJ (2010). Mammalian autophagy: core molecular machinery and signaling regulation. Curr Opin Cell Biol.

[11] Lamark T, Johansen T (2021). Mechanisms of selective autophagy. Annu Rev Cell Dev Biol.

[12] Nähse V, Schink KO, Stenmark H (2024). ATPase-regulated autophagosome biogenesis. Autophagy.

[13] Zhen Y, Stenmark H (2023). Autophagosome biogenesis. Cells.

[14] Kannangara AR, Poole DM, McEwan CM, Youngs JC, Weerasekara VK, Thornock AM, Lazaro MT (2021). BioID reveals an ATG9A interaction with ATG13‐ATG101 in the degradation of p62/SQSTM1‐ubiquitin clusters. EMBO Rep.

[15] Thorne RF, Yang Y, Wu M, Chen S (2022). TRIMming down autophagy in breast cancer. Autophagy.

[16] Alers S, Wesselborg S, Stork B (2014). ATG13. Autophagy.

[17] Obara K, Ohsumi Y (2011). Atg14: a key player in orchestrating autophagy. Int J Cell Biol.

[18] Hurley JH, Young LN (2017). Mechanisms of autophagy initiation. Annu Rev Biochem.

[19] Chang HC, Tao RN, Tan CT, Wu YJ, Bay BH, Yu VC (2021). The BAX-binding protein MOAP1 associates with LC3 and promotes closure of the phagophore. Autophagy.

[20] Alam JM, Maruyama T, Noshiro D, Kakuta C, Kotani T, Nakatogawa H, Noda NN (2024). Complete set of the Atg8-E1-E2-E3 conjugation machinery forms an interaction web that mediates membrane shaping. Nat Struct Mol Biol.

[21] Klionsky DJ, Abdel-Aziz AK, Abdelfatah S, Abdellatif M, Abdoli A, Abel S, Abeliovich H (2021). Guidelines for the use and interpretation of assays for monitoring autophagy (4th edition). Autophagy.

[22] Liu E, Lopez Corcino Y, Portillo JAC, Miao Y, Subauste CS, Adams JH (2016). Identification of signaling pathways by which CD40 stimulates autophagy and antimicrobial activity against
*Toxoplasma gondii* in macrophages. Infect Immun.

[23] Subauste CS, Andrade RM, Wessendarp M (2007). CD40-TRAF6 and autophagy-dependant anti-microbial activity in macrophages. Autophagy.

[24] Liu B, Su Y, Li T, Yuan W, Mo X, Li H, He Q (2015). CMTM7 knockdown increases tumorigenicity of human non-small cell lung cancer cells and EGFR-AKT signaling by reducing Rab5 activation. Oncotarget.

[25] Xia D, Qu L, Li G, Hongdu B, Xu C, Lin X, Lou Y (2016). MARCH2 regulates autophagy by promoting CFTR ubiquitination and degradation and PIK3CA-AKT-MTOR signaling. Autophagy.

[26] Elgueta R, Benson MJ, De Vries VC, Wasiuk A, Guo Y, Noelle RJ (2009). Molecular mechanism and function of CD40/CD40L engagement in the immune system. Immunol Rev.

[27] Zhang T, He YM, Wang JS, Shen J, Xing YY, Xi T (2011). Ursolic acid induces HL60 monocytic differentiation and upregulates C/EBPβ expression by ERK pathway activation. Anti-Cancer Drugs.

[28] Gao M, Zhao LR (2018). Turning death to growth: hematopoietic growth factors promote neurite outgrowth through MEK/ERK/p53 pathway. Mol Neurobiol.

[29] Zhong S, Zhang C, Johnson DL (2004). Epidermal growth factor enhances cellular TATA binding protein levels and induces RNA polymerase I- and III-dependent gene activity. Mol Cell Biol.

[30] Santalucia T, Sanchezfeutrie M, Felkin L, Bhavsar P, Barton P, Zorzano A, Yacoub M (2005). Phenylephrine requires the TATA box to activate transcription of GLUT1 in neonatal rat cardiac myocytes. J Mol Cell Cardiol.

[31] Numakawa T, Odaka H, Adachi N, Chiba S, Ooshima Y, Matsuno H, Nakajima S (2018). Basic fibroblast growth factor increased glucocorticoid receptors in cortical neurons through MAP kinase pathway. Neurochem Int.

[32] Grazia GA, Bastos DR, Villa LL (2023). CD40/CD40L expression and its prognostic value in cervical cancer. Braz J Med Biol Res.

[33] Jung CH, Jun CB, Ro SH, Kim YM, Otto NM, Cao J, Kundu M (2009). ULK-Atg13-FIP200 complexes mediate mTOR signaling to the autophagy machinery. Mol Biol Cell.

[34] Ganley IG, Lam DH, Wang J, Ding X, Chen S, Jiang X (2009). ULK1·ATG13·FIP200 complex mediates mTOR signaling and is essential for autophagy. J Biol Chem.

[35] Hosokawa N, Hara T, Kaizuka T, Kishi C, Takamura A, Miura Y, Iemura S (2009). Nutrient-dependent mTORC1 association with the ULK1-Atg13-FIP200 complex required for autophagy. Mol Biol Cell.

[36] Wang H, Guo M, Wei H, Chen Y (2023). Targeting p53 pathways: mechanisms, structures and advances in therapy. Signal Transduct Target Ther.

[37] Xie K, Liu L, Wang M, Li X, Wang B, Yin S, Chen W (2023, 55: 623–632). IMPA2 blocks cervical cancer cell apoptosis and induces paclitaxel resistance through p53-mediated AIFM2 regulation. Acta Biochim Biophys Sin.

[38] Kenzelmann Broz D, Spano Mello S, Bieging KT, Jiang D, Dusek RL, Brady CA, Sidow A (2013). Global genomic profiling reveals an extensive p53-regulated autophagy program contributing to key p53 responses. Genes Dev.

[39] Wilson EN, Bristol ML, Di X, Maltese WA, Koterba K, Beckman MJ, Gewirtz DA (2011). A switch between cytoprotective and cytotoxic autophagy in the radiosensitization of breast tumor cells by chloroquine and vitamin D. Horm Cancer.

[40] Ondrej M, Cechakova L, Durisova K, Pejchal J, Tichy A (2016). To live or let die: Unclear task of autophagy in the radiosensitization battle. RadioTher Oncol.

